# Reconstruction of the Rat Sciatic Nerve by Using Biodegradable and Non-Biodegradable Conduits

**DOI:** 10.17691/stm2020.12.5.05

**Published:** 2020-10-28

**Authors:** A.G. Velichanskaya, D.A. Abrosimov, M.L. Bugrova, A.V. Kazakov, E.V. Pogadaeva, A.M. Radaev, N.V. Blagova, T.I. Vasyagina, I.L. Ermolin

**Affiliations:** Associate Professor, Department of Histology, Cytology, and Embryology; Privolzhsky Research Medical University, 10/1 Minin and Pozharsky Square, Nizhny Novgorod, 603005, Russia;; Senior Lecturer, Department of Histology, Cytology, and Embryology; Privolzhsky Research Medical University, 10/1 Minin and Pozharsky Square, Nizhny Novgorod, 603005, Russia;; Associate Professor, Head of the Department of Electron Microscopy, Central Research Laboratory; Privolzhsky Research Medical University, 10/1 Minin and Pozharsky Square, Nizhny Novgorod, 603005, Russia;; Researcher, Research Laboratory, Clinic of Cardiology, Angiology, and Intensive Care; Saarland University, Saarbrücken Campus, Saarbrücken, 66123, Germany; Senior Laboratory Assistant, Department of Histology, Cytology, and Embryology; Privolzhsky Research Medical University, 10/1 Minin and Pozharsky Square, Nizhny Novgorod, 603005, Russia;; Associate Professor, Department of Histology, Cytology, and Embryology; Privolzhsky Research Medical University, 10/1 Minin and Pozharsky Square, Nizhny Novgorod, 603005, Russia;; Senior Lecturer, Department of Histology Cytology, and Embryology; Privolzhsky Research Medical University, 10/1 Minin and Pozharsky Square, Nizhny Novgorod, 603005, Russia;; Senior Researcher, Department of Electron Microscopy, Central Research Laboratory; Privolzhsky Research Medical University, 10/1 Minin and Pozharsky Square, Nizhny Novgorod, 603005, Russia;; Professor, Head of the Department of Histology, Cytology, and Embryology Privolzhsky Research Medical University, 10/1 Minin and Pozharsky Square, Nizhny Novgorod, 603005, Russia;

**Keywords:** peripheral nerve, nerve regeneration, conduit for nerve repair, Reperen, Tissucol Kit

## Abstract

**The aim of the study** was to compare two types of conduits made of either non-resorbable Reperen or resorbable Tissucol for their effects on the regeneration of the rat sciatic nerve under conditions of stump diastasis.

**Materials and Methods.:**

The experiments were carried out using outbred white male rats of the reproductive age (n=14). The animals were divided into three groups: group 1, intact (n=5), used for studying the morphology of the sciatic nerve; group 2 (n=4) — nerve plastic surgery was performed using a conduit made of non-resorbable Reperen; group 3 (n=5) — surgery was performed using a conduit made of resorbable Tissucol. The animals were anesthetized with isoflurane. After a complete transection of the sciatic nerve in the middle third of the thigh, its stumps were inserted into a conduit of an internal diameter of 2 mm and a length of 10 mm, filled with saline. Diastasis of 5 mm in length was created by spreading the nerve ends and securing the epineurium to the tube edges with 8/0 polypropylene sutures. A total count of myelinated nerve fibers was performed in the area of repair (tubulation) and the distal part of the nerve; the formation of connective tissue sheaths was assessed 14 weeks after the operation.

**Results.:**

According to the morphological assessment, both types of conduits (resorbable and non-resorbable) caused the similar number of fibers to restore in the distal part of the repaired nerve; clinical characteristics of the animals in both groups were close to each other and to the norm.

**Conclusion.:**

The results allow us to consider the conduit made of non-resorbable Reperen as a device promising for neuroplasty along with the resorbable conduit made of Tissucol.

## Introduction

Peripheral nerve injury leads to a decrease or loss of functions of the injured limb and may disable the injured person. Restoration of the nerve and its structure is one of the main areas of research in neurosurgery and neuro-histology [1]. Suturing of nerve trunks is inevitably accompanied by the formation of connective tissue scars, which do not allow for complete restoration of limb functions [2–4]. The primary method of microsurgery for diastasis of the nerve stumps is autoplasty by using parts of other peripheral nerves [5–13]. During this operation, the blood supply to the recovering nerve is disrupted, which affects the nerve regeneration and promotes the formation of connective tissue scars [14–20]. In addition, autoplasty is accompanied by soreness of the donor sites, impaired sensitivity, and formation of neuromas [21–24].

In preclinical neuro-histology, a radically novel approach has recently emerged: the so-called tubulation of the nerve trunk using conduits — tubes that serve substrates for nerve repair [25, 26]. Under conditions of diastasis, such a substrate can also contain the filler that stimulates nerve regeneration. In addition, a conduit prevents the ingrowth of surrounding connective tissue into the area of injury and thus limits the formation of a connective tissue scar, which may interfere with the penetration of growing nerve fibers into the distal stump [21, 27].

There are reports on using unfilled biodegradable and non-biodegradable tubes to study the effect of various fillers on nerve regeneration [5, 23, 24, 27–32]. Good results were obtained with biodegradable conduits, including those made of Tissucol fibrin glue [27, 31, 32]. In neurosurgery, the biodegradable biocompatible Reperen was used for plastic surgery on the dura mater; this material keeps the brain structures intact and prevents their adhesions to the surrounding tissues [33]. A Reperen plate was used to isolate the trigeminal nerve root by wrapping it to stop pain generated by arterial pulsation [34]. However, tubes made of non-biodegradable materials are rarely used in plastic surgery for peripheral nerve reconstitution, and preference is given to biodegradable tubes.

In this regard, **the aim of the study** was to compare two types of conduits shaped as uniform tubes made of either non-resorbable Reperen or resorbable Tissucol for their effects on the regeneration of the rat sciatic nerve under conditions of stump diastasis.

## Materials and Methods

The experiments were carried out using white outbred male rats of reproductive age (n=14), weighing 350–400 g, in accordance with the Good Laboratory Practice (Russia, 2016), with the regulations of the Guide for the Care and Use of Laboratory Animals (National Research Council, 2011), and with the ethical principles of the European Convention for the Protection of Vertebrate Animals used for Experimental and Other Scientific Purposes (Strasbourg, 2006). The study protocol was approved by the Ethics Committee of the Privolzhsky Research Medical University.

The animals were divided into three groups: group 1, intact (n=5), used for studying the morphology of an normal sciatic nerve; group 2 (n=4) — nerve plastic surgery was performed using a conduit made of non-resorbable Reperen; group 3 (n=5) — surgery was performed using a conduit made of resorbable Tissucol.

The animals were anesthetized with isoflurane using a Zoomed minor vet anesthesia machine (Beijing Read Eagle Technology Co., Ltd., China) and an Armed 7F-3L oxygen concentrator (Jiangsu Yuyue Medical Equipment and Supply Co., Ltd., China). The sciatic nerve of the right hind limb was accessed by cutting the skin along the projection of the femur and then bluntly dissecting the muscle fascia. The isolated nerve was cut transversely in the middle third of the thigh; the stumps were inserted into a saline-filled conduit tube of an inner diameter of 2 mm and a length of 10 mm. Diastasis of 5 mm was created by spreading the ends of the transected nerve, inserting these ends into the conduit to a length of 2.5 mm on each side, and fixing the epineurium to the tube using interrupted sutures of 8/0 polypropylene.

Two types of conduits were used in the experiments; those made of Reperen (Icon Lab, Russia) were supplied as ready-made sterile tubes; the fibrin glue conduits were prepared in this lab using a Tissucol Kit (Baxter, Austria) according to the well-known method [23, 27, 28].

For morphological analysis, semi-thin (0.5 μm) transverse sections of the sciatic nerve were prepared using a Leica EM UC7 ultramicrotome (Leica, Germany) and stained with methylene blue and fuchsine. The growing nerve at the site of tubulation and the distal stump at 1 mm from the conduit were probed in 14 weeks after the operation.

The material was fixed in a 2.5% solution of glutaraldehyde in phosphate buffer (pH 7.4) and postfixed in a 1% solution of osmium tetroxide, followed by embedding in the Epon-Araldite resin mixture. Photographs of the resulting slides were taken with a Nikon Eclipse 80i light microscope (Nikon DS-Fi1 camera; Nikon, Japan) with an eyepiece magnification of ×10 and objectives of ×10 and ×20.

Morphometric data were processed with the NIS-Elements BR 4.0 software. We determined the total number of regenerated myelinated nerve fibers and then assigned them into size groups, based on the nerve fiber diameter according to [35]: small (less than 4 μm in diameter), medium (4–7 μm), and large (>7 μm). We also estimated the total area of the transverse section of the nerve, the area of the nerve fiber bundles, and the area of the epineurium.

**Statistical processing** of the data was performed with the Statistica 10.0 software using both the basic and nonparametric methods. Statistical significance of the differences between the compared values was assessed using the Mann–Whitney test at p<0.05.

## Results

### Morphology of the intact sciatic nerve.

**Figure 1:**
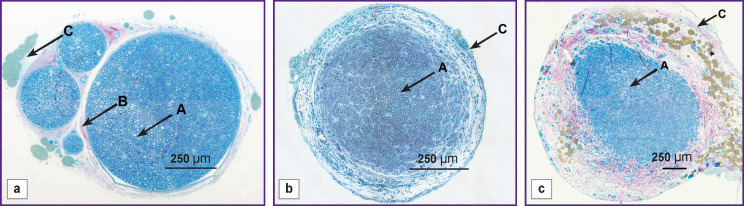
Cross-section of the rat sciatic nerve: (a) in an intact animal; (b) regenerated in a conduit from Reperen after 14 weeks; (c) regenerated in a conduit from Tissucol after 14 weeks; *A* — nerve fibers, *B* — perineurium, *C* — epineurium; staining with methylene blue and fuchsine

 Normally, the area of interest in the sciatic nerve consists on average of 4 nerve bundles, each surrounded by perineurium. The epineural membrane is thin, with a moderately developed adipose tissue (Figure 1 (a)). The total area of the rat sciatic nerve is 1,365,632.0±180,727.5 μm^2^; the area of the nerve without epineurium was 782,832.3±160,703.0 μm^2^, and the area of the epineurium — 582,799.5±110,457.5 μm^2^.

Analysis of size distribution of myelinated fibers showed that large fibers (d>7 μm) prevailed (57.7%), the medium size fibers (4<d<7 μm) accounted for 37.3%, and the small ones (d<4 μm) represented 5.7% of the total number of nerve fibers.

### Morphology of the growing sciatic nerve at the site of tubulation.

 Fourteen weeks after the tubulation, there were notable morphological differences between the injury site treated with the Reperen-based conduit and that treated with Tissucol.

**Figure 2:**
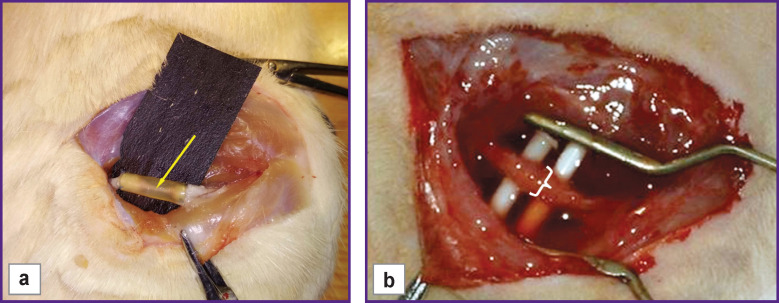
The rat sciatic nerve upon 14 weeks of regeneration: (a) in a Reperen-based conduit; (b) in a Tissucol-based conduit (marked with a staple)

With Reperen, the post-injury nerve segment was thinner than its distal segment or the intact nerve. The regenerating nerve trunk was located inside a non-resorbable tube and did not adjoin its inner surface (Figure 2 (a)). It contained one bundle of nerve fibers (Figure 1 (b)). The average cross-sectional area of the nerve with epineurium was 1.7-fold smaller than normal; and that without epineurium — 2.1-fold smaller than normal. The cross-sectional area of the epineurium was 1.37-fold smaller than that in the intact nerve. In the size groups, the mid-sized myelinated nerve fibers prevailed, accounting to 53.7% of the total number of fibers (see the Table). The respective figure for small fibers was 35.1% and that for large ones — 11.2%. In total, the number of myelinated nerve fibers in all size groups was significantly different from the norm.

**Table:** The number of myelinated nerve fibers in the sciatic nerve tubulated with either biodegradable or non-biodegradable conduit

Size groups of myelinated nerve fibers	Norm	Injury area		Distal stump	
		Reperen	Tissucol	Reperen	Tissucol
Large (d>7 μm)	2612±212	1070±287*^+^	840±438*^v^	156±78*^#^	314±106*°
Medium (4<d<7 μm)	1710±397	5140±1022*^+^	4910±1412*^v^	2265±807*^#^	2330±585°
Small (d<4 μm)	259±99	3356±514*^+^°	10,809±967*^#v^	6118±1040*^#^	4918±225*°
Total number	4582±324	9567±1576*°	16,560±1888*^#v^	8540±1340*	7562.5±1453.35*°

Notes: the differences are statistically significant (p<0.05): * between the data samples and the norm; ^#^ between the data samples and the Reperen-treated zone; ^+^ between the data samples and the distal stump in the Reperen-treated nerve; ° between the data samples and the Tissucol-treated zone; ^v^ between the data samples and the distal stump in the Tissucol-treated nerve.

The conduit made of Tissucol was completely resorbed 14 weeks after the surgery. The regenerated area (Figure 2 (b)) was much thicker than that in the Reperen-based conduit. No perineurium was detected, and the nerve fibers were presented as one bundle (Figure 1 (c)). The epineurium was greatly thickened and had a loose structure. Accordingly, the total area of the nerve section increased significantly and exceeded the intact values by 2.68 times. In addition, the average area of the epineurium section was almost 4.0 times larger than that in the intact sciatic nerve. The average nerve area without epineurium was 1.76 times larger than the norm.

Total number of myelin fibers at the site of injury increased 3.6-fold the normal level (see the Table). Among the size groups, small myelin fibers prevailed, accounting for 65.3% of the total. The presence of medium size fibers was 29.6%, which was close to the norm, and the percentage of large fibers was only 5.1%.

Additional differences between the conduits were found at the end of the experiment. No adhesions were observed around the Reperen tube. It was covered with a layer of thin connective tissue and could easily be detached from the surrounding tissue. With Tissucol, following conduit resorption the newly formed nerve was difficult to separate from the surrounding tissues due to pronounced adhesions.

### Morphology of the distal segment of the sciatic nerve.

**Figure 3:**
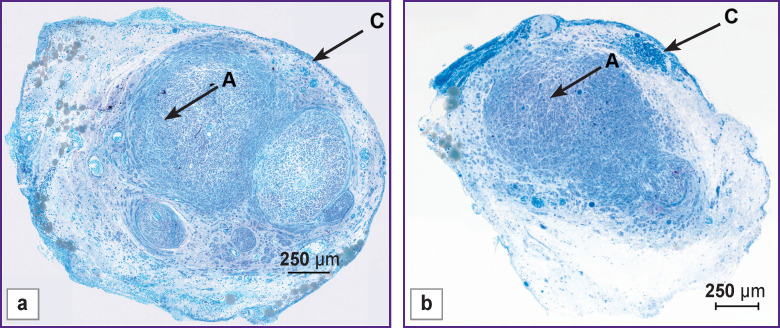
Regenerated rat sciatic nerve 1 mm distal to the conduit (after 14 weeks): (a) with Reperen; (b) with Tissucol; *A* — nerve fibers, *C* — epineurium; staining with methylene blue and fuchsine

 By the end of the experiment (week 14), the transected nerve tubulated with Reperen showed the morphological characteristics preserved when probed at 1 mm distal of the tube edge (Figure 3 (a)). However, the total cross-sectional area of the nerve trunk exceeded the normal values 2–3-fold due to the growing epineurium. In this part of the dissected nerve, the cross-sectional area of nerve bundles decreased compared to the intact nerve. The size distribution showed the greatest prevalence of small myelin fibers (71.0%), followed by mid-sized fibers (26.5%) and then large fibers (1.8%).

After tubulation with Tissucol, a single bundle of nerve fibers surrounded by a wide loose epineurium was observed in the distal stump by the end of the experiment. The perineum was not expressed. The cross-sectional area of the nerve in this distal segment exceeded the area of the intact nerve due to the overgrown epineurium. The total number of myelinated nerve fibers was almost twice higher than that in the intact nerve. The number of large myelin fibers was 4.1%, medium — 30.8%, and small — 65.0%.

### Clinical indicators of the recovering injured limb.

 In intact rats, plantar flexion has a range of motion close to 180° [36]. If the sciatic nerve is damaged, a reduced range can indicate a joint contracture.

**Figure 4:**
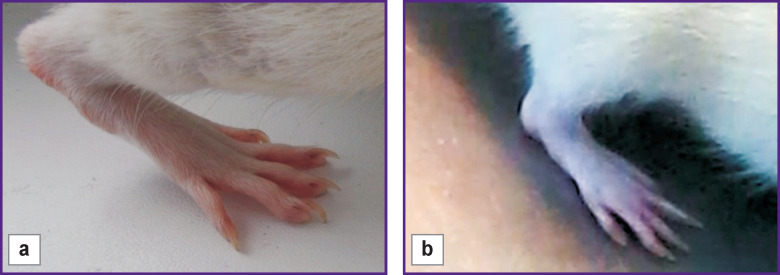
Position of the right hind limb after 14 weeks of nerve regeneration: (a) with a conduit made of Reperen; (b) with a conduit made of Tissucol

In this study, we used the following indicators of the right hind limb injury: 1) range of motion of the ankle; 2) limb position; 3) inflammation of the ankle joint with the appearance of trophic ulcers; 4) the number of toes. According to these criteria, both types of conduits for nerve regeneration showed similar results by the end of the experiment. The ankle joint extension angle averaged at 170°, no inflammation or thickening of the joint was observed. When walking, the animals leaned on the foot and straightened the toes of the injured limb (Figure 4).

## Discussion

In the present experiment, the morphological characteristics of the sciatic nerve were studied. We determined the total number of myelinated nerve fibers and their distribution by three size groups; we also assessed the cross-section area of the sciatic nerve with and without the epineurium, as well as the epineurium itself. The obtained size distribution of myelinated nerve fibers differs from the results of others [35], who counted nerve fibers by vision fields.

In both experimental groups, 14 weeks after surgery, in the area of tubulation, there was a significant increase in the total number of nerve fibers compared to the intact nerve. According to some reports [37, 38], this fact may be associated with the multiplication of regenerating nerve fibers during the transition from the proximal stump to the area of injury. In this location, myelinated nerve fibers become thinner due to their multiplication. The similar finding was reported by others [39], who used different types of conduits and analyzed the nerve fibers 8 weeks after surgery. Notably, the fibrin conduit (Tissucol) attracted a greater number of small myelinated nerve fibers, which grow into the epineurium that replaced the Tissucol tube.

The morphological differences in the regenerating nerve under the two compared types of tubulation are due to the fundamental difference in the structure of conduits. Fibrin glue contains fibrinogen and fibronectin, which are components of the extracellular matrix and are capable of stimulating the proliferation of connective tissue [40, 41]. Apparently, the pronounced proliferation of epineurium in the Tissucol-based conduit is promoted by fibrin that plays the role of acellular matrix for the newly-formed epineurium. In addition, adhesions were noted between the conduit and the surrounding tissues, which impeded the natural mobility of the nerve in its bed (tunnel syndrome) and could have a negative effect on the nerve regeneration.

Reperen is a spatially cross-linked polymer made of methacrylic oligomers with no micropores. The material is highly bio-compatible, bio-stable, and inert in relation to body components; due to that, it does not undergo bio-aging and does not cause adhesion. Reperen was used as a surface pad to grow fibroblast monolayers; in this setting, no 3D formation of connective tissue occurred and adhesions did not develop [33]. In addition, Reperen prevents the penetration of actively dividing connective tissue into the area of injury.

Fourteen weeks after tubulation with Tissucol, a bundle of nerve fibers with no clear boundaries, surrounded by a wide loose epineurium, was observed in the distal stump. The perineural membrane is not expressed. A similar structure in the distal portion of a reconstructed rat nerve was reported by others [27]; there, the transected nerve was tubulated with a fibrin conduit at diastasis of 1 cm.

Here, in both experimental groups, prevalence of small nerve fibers was detected in the zone of transition from the area of injury to the distal stump; in parallel, the number of large and medium-sized nerve fibers decreased. In addition, in the case of fibrin, we noted a reduction in the total number of nerve fibers at the spot of their transition from the tubulation site to the distal segment. Others also noted a similar decrease 8 weeks after the operation [39].

The large number of small nerve fibers in the area tubulated with a Tissucol conduit can be explained by the excessive growth of epineurium and the associated vascularization. In this case, small nerve fibers are attracted to innervate the epineurium-associated structures and remain within the area of injury, forming their connections with new targets there. This may be the reason for the further reduction in the numbers of fibers during their transition to the distal stump. Similar observations were reported earlier [37].

The increase in the total number of nerve fibers in the distal segment occurs in both experimental groups. However, with Repren, the total number of myelinated nerve fibers does not change when passing from the tubulation zone to the distal segment and but it decreases with Tissucol. Presumably, there is no multiplication of conductors when the nerve crosses the distal border zone, in contrast to the proximal one.

In using Reperen and Tissucol, we noted that the clinical parameters of the injured limb correlated with the morphology of the distal part rather than the zone of tubulation of the regenerated sciatic nerve. The nerve structure in the area of tubulation did not correlate with the clinical picture of the operated limb. However, the quantitative characteristics of impulse conductors in the distal part of the nerve showed good correlations with the clinical parameters of the operated limb when using either type of the conduits. We found no relevant data on this specific issue in the literature. In some studies investigating the effects of conduits on the peripheral nerve [27, 39], morphology of the distal part of the damaged nerve was also assessed, but no correlation analysis between the morphological and clinical parameters was described.

## Conclusion

The use of Reperen and Tissucol for peripheral nerve plastic surgery showed principally different ways of regeneration of either nerve conductors or nerve sheaths. The structure of the repaired nerve in the area of injury differed significantly from the norm in both cases. However, the quantitative indicators of the distal part were practically the same when either of the conduits was used; moreover, the clinical pictures were close to normal in both cases. Along with this consistency, the two materials significantly differ from each other. Specifically, the fibrin-based conduit undergoes resorption followed by a pronounced adhesive process, which can lead to the development of the “tunnel syndrome”. The Reperen conduit is not resorbed, yet the adhesion process in the tubulation area is limited, which provides for sufficient mobility of the restored nerve in its bed. Reperen-made conduits are contraindicated for use in a growing body. Nevertheless, in emergency cases of nerve rupture, the use of Reperen is a faster and safer way of repair, since tubes made of this material are available at different diameters and supplied in sterile packages. In contrast, a Tissucol conduit must be made at the spot immediately before the operation, which requires more time and special equipment.

The present results allow us to consider conduits made of non-resorbable Reperen as promising for neuroplasty of peripheral nerves along with resorbable conduits made of Tissucol.
